# An Inter-University Virtual Geriatric Case Competition to Build Interprofessional Collaboration Skills

**DOI:** 10.1093/geroni/igab046.2809

**Published:** 2021-12-17

**Authors:** Kristine Talley, Marla Berg-Weger, Devita Stallings, Teresa Schicker, Laura Pesja, Jean Wyman

**Affiliations:** 1 University of Minnesota, Minneapolis, Minnesota, United States; 2 Saint Louis University, Saint Louis, Missouri, United States; 3 Saint Louis University, St. Louis, Missouri, United States

## Abstract

Developing positive learning experiences in team-based geriatric care is challenging. This presentation will highlight an inter-University geriatric case competition for developing interprofessional competencies in health professional students sponsored by the Geriatric Workforce Enhancement Programs at Saint Louis University and the University of Minnesota. The virtual competition involved teams of 4-5 undergraduate and graduate students from multiple health professions who designed a comprehensive care plan using a simulated complex geriatric patient case. Students were assigned to an interprofessional team with a faculty or community expert coach, attended an orientation, and developed a 20-minute recorded presentation. A panel of judges rated team presentations using a scoring rubric based on the Core Competencies for Interprofessional Collaborative Practice. Local competitions included a first and semi-final round, with the winning teams presenting at the inter-university competition held via live videoconferencing that involved a question-and-answer session. Prizes were given to the top teams. Students, coaches, and judges completed evaluation surveys focused on satisfaction with the competition format/procedures and achievement of interprofessional competencies. Twenty-one teams and 117 students from 12 disciplines participated. Team scores ranged from 2.2 to 4.3 (overall mean 3.1) on a 1-5 scale. Judge, coach, and student evaluations were positive, indicating students learned valuable lessons in group dynamics, team-based care, and geriatric care. Most students (82%) preferred the virtual competition format or had no preference on format. The virtual case competition provided a positive, engaging experience to introduce health professional students to geriatric team-based care and develop their readiness for collaborative practice.

